# Unraveling Stuttering Through a Multi-Omics Lens

**DOI:** 10.3390/life15101630

**Published:** 2025-10-19

**Authors:** Deyvid Novaes Marques

**Affiliations:** Department of Genetics, University of São Paulo (USP), Piracicaba 13418-900, São Paulo (SP), Brazil; deyvidnovaes@gmail.com

**Keywords:** stuttering, developmental stuttering, genetics, genomics, multi-omics, omics, phenomics, speech disorders, genome-wide association studies, biomarkers

## Abstract

Stuttering, a complex and multifactorial speech disorder, has long presented an enigma regarding its etiology. While earlier approaches often emphasized psychosocial influences, historical clinical and speech-language strategies have considered multiple contributing factors. By integrating genomic, transcriptomic and phenomic evidence, the ongoing research illustrates how functional genomics can unravel the biological architecture of complex speech disorders. In particular, advances in omic technologies have unequivocally positioned genetics and underlying biological pathways at the forefront of stuttering research. I have experienced stuttering and lived with it since my early childhood. This perspective article presents findings from omic studies, highlighting relevant aspects such as gene discoveries, implicated cellular mechanisms, and the intricate genetic architecture of developmental stuttering. As a person who stutters, I offer an intimate perspective on how these scientific insights are not merely academic but profoundly impactful for the affected community. A multi-omic integration strategy, combining large-scale genetic discovery with deep phenotyping and functional validation, is advocated to accelerate understanding in this field. Additionally, a bibliometric analysis using an international database was conducted to map trends and identify directions in stuttering research within the omic context. Ultimately, these scientific endeavors hold the potential to inform not only personalized interventions but also critical policy and regulatory changes, enhancing accessibility, support, and the recognized rights of people who stutter.

## 1. Stuttering Unraveled: Context and the Omics Field

Developmental stuttering, also known as childhood-onset fluency disorder, is a complex and multifactorial speech disorder characterized by involuntary repetitions of sounds, syllables, or words, as well as prolongations and blocks, without apparent neurogenic or psychogenic impairment, and not associated with conditions such as brain injury [1,2]. It is a speech fluency disorder distinct from primary language impairments, although these conditions may share some genetic influences and are being investigated in different countries. Stuttering is also recognized as a neurodevelopmental disorder marked by atypical formation of the networks responsible for speech motor planning and execution. These networks must interact in highly coordinated ways with neural systems responsible for language, as well as broader cognitive and emotional processes [3]. Disruptions in these interactions may contribute to the emergence and persistence of stuttering, highlighting the complex neurobiological basis of the disorder. Affecting people globally, persistent developmental stuttering (PDS) typically emerges in early childhood and persists into adulthood for a significant portion of those affected [4]. Within this context, unlike acquired stuttering, which can result from events such as neurological injury, some individuals experience the form of stuttering that is a primary speech disorder, typically emerging during development and, in many cases, not associated with aspects related to identifiable psychological trauma or not primarily caused by such factors. However, it can coexist with other neurological conditions or comorbidities, highlighting the complexity of its clinical presentation [2,5]. Despite its prevalence and significant impact on daily life, the precise etiology of this developmental stuttering remains elusive, leading to persistent challenges in diagnosis, intervention, and societal understanding.

Historically, explanations for stuttering largely centered on psychological or environmental factors. However, decades of research, particularly in genetics, have firmly established a significant biological predisposition to PDS [6]. The advent of high-throughput ‘omics’ technologies, encompassing several approaches such as genomics, transcriptomics, and phenomics, has revolutionized the capacity to probe the biological underpinnings of complex disorders like PDS on an unprecedented scale.

**Figure 1:**
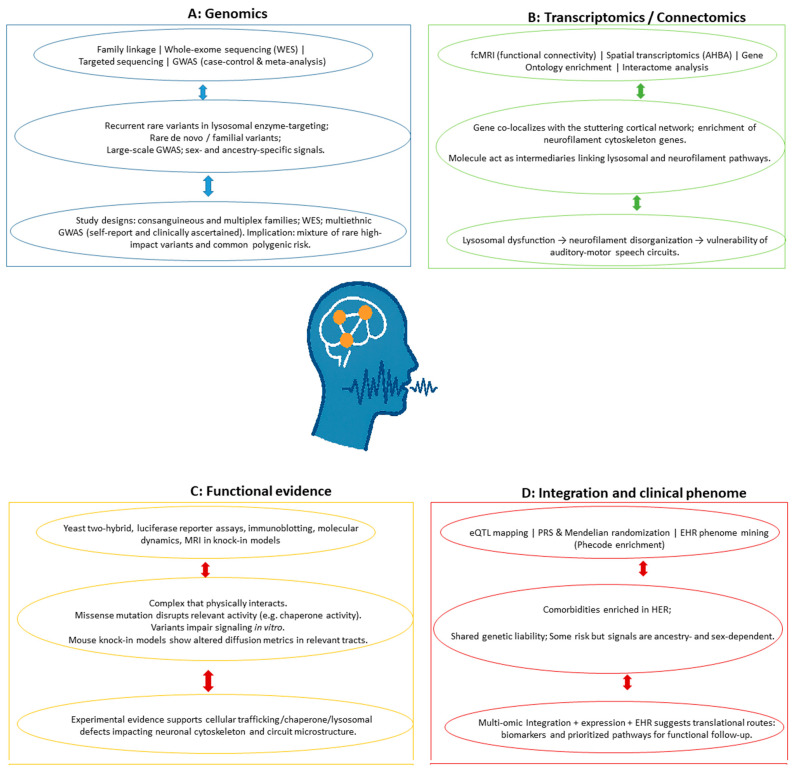
Overview of research topics and selected omic-related approaches to developmental stuttering. (**Panel A**) covers genomic study designs. (**Panel B**) presents transcriptomic and connectomic focus that spatially localize some relevant topics. (**Panel C**) depicts functional genomics evidence. (**Panel D**) displays omic integration approaches and clinical phenotypes. Arrows indicate potential research associations. Abbreviations and more detailed information are presented in the main text.

I have experienced stuttering and lived with it since my early childhood. From my dual perspective as a PhD scientist and a person who stutters, I recognize the immense potential of these scientific advancements to move beyond descriptive observations, elucidate precise biological pathways, reduce stigma, and ultimately transform support for the stuttering community. This article provides information regarding omic findings in stuttering research, with a particular focus on developmental stuttering and PDS, offering a roadmap and perspective for future research and emphasizing the crucial link between scientific discovery, multi-omic focus and improved quality of life for people who stutter. Furthermore, this article situates stuttering research within the broader context of functional genomics, demonstrating how speech-related traits can inform genotype-to-phenotype mapping and multi-omic integration strategies. This perspective is further enriched by a bibliometric analysis based on an international database, highlighting publications within this research topic and presented throughout the manuscript. An overview of key approaches employed in developmental stuttering and associated research aspects within the omic field is presented in Figure 1.

## 2. Genomic Landscapes: From Linkage Scans to Genome-Wide Associations

Some genetic investigations into PDS primarily relied on family-based linkage studies, which sought to identify broad chromosomal regions shared among affected family members. Relevant approaches paved the way by providing insights into the heritable nature of stuttering and pointing to multiple loci potentially involved in stuttering. Genome-wide linkage scans in 68 families identified chromosome 18 as a potential predisposing locus for PDS [7]. Later, a comprehensive scan using multiple single nucleotide polymorphisms (SNPs) across 100 families revealed sex-specific genetic effects, with significant linkage on chromosome 7 in males and chromosome 21 in females, emphasizing the role of sex and the polygenic complexity of stuttering [8].

Further investigations in diverse populations continued to expand the genetic map within the stuttering context. Genome-wide linkage and association analyses in a Hutterite founder population identified several nominally significant regions on chromosomes 13, 3, 9, 5, 15, and 1, supporting its polygenic basis [9]. A study in a highly consanguineous Pakistani family revealed a novel autosomal recessive locus on chromosome 3q13.2–3q13.33 [10], while research in a large Cameroonian family identified additional loci on chromosomes 2p, 3p, 14q, and a distinct region of 15q [11]. More recently, a linkage scan in Brazilian families detected a new dominant locus on chromosome 10q21 [12]. The consistent finding of genetic heterogeneity across these studies underscores that stuttering is influenced by multiple genes with varying effects, rather than a single highly penetrant allele [9,11,12].

The shift from broad linkage studies to Genome-Wide Association Studies (GWAS) enabled the identification of specific common genetic variants in stuttering. Relevant GWAS-related investigations reported multiple candidate genes with roles in neural development, function, and behavior [6]. More recent trans-ancestry and population-based GWAS confirmed additional significant and suggestive loci, highlighting the polygenic and complex nature of developmental stuttering and the distinction between family-based and population-level genetic influences [13,14].

## 3. Targeted Gene Discovery and Pathway Elucidation: Deepening Molecular Insights Within an Omic Context

Beyond identifying broad genetic regions, advances in omics-related research have enabled the discovery of specific genes and molecular pathways implicated in stuttering. A relevant study identified rare mutations affecting the lysosomal enzyme–targeting pathway, which were specifically associated with nonsyndromic PDS rather than typical mucolipidosis symptoms [15]. Subsequent large-scale analyses confirmed that these rare coding variants account for a substantial fraction of PDS cases and are functionally distinct from variants causing mucolipidoses [16]. A whole-exome sequencing (WES) study has revealed additional cellular mechanisms contributing to PDS, including deficits in intracellular trafficking and endosomal transport [17]. Furthermore, integration of genetic data with neuroimaging highlighted disruptions in neurofilament organization and cortical networks, suggesting potential links between genetic variants and altered brain connectivity in stuttering [18].

More recent stuttering-focused investigations continue to broaden our understanding, prospecting or identifying variants affecting diverse pathways, including synaptic function, dopamine signaling, Wingless and Int-1 signaling, interferon pathways, and protein chaperoning [2,19,20,21]. These omic-related studies underscore the complex and heterogeneous genetic architecture of developmental stuttering, revealing multiple molecular pathways that converge and highlight the potential for future functional studies to unravel the mechanistic links between gene variants and stuttering phenotypes.

## 4. Phenomics: Characterizing the Clinical Landscape for Genetic Discovery

While genomic approaches focus on discovering genetic variants, phenomics, the large-scale study of phenotypes, is also relevant for robust omics research. Deep and standardized phenotyping allows for the precise definition of affected individuals and the identification of associated clinical features, which can then be linked to genetic data. A significant advancement in this area involved the development of a multi-step phenotyping approach to identify PDS cases and their associated comorbidities within electronic health records (EHRs) [5]. Such a study utilized a phecode enrichment analysis, identifying a diverse array of significantly enriched comorbidities, including developmental delays, speech and language disorders, hearing loss, sleep disorders, and surprisingly, conditions related to the atopic triad (e.g., allergies, dermatitis) and increased overall childhood medical conditions [5]. The development of a Phenome Risk Classifier (PheRC), a machine learning model, offers a scalable method to expand the number of phenotypically similar stuttering cases for future large-scale genetic analyses, such as GWAS [5,14]. This systematic characterization of the phenome associated with stuttering is vital for building powerful cohorts for multi-omic investigations.

## 5. A Multi-Omic Future: Integrating Layers for Comprehensive Understanding

To illustrate and present important advances in the field in more detail, Table 1 provides relevant examples and related information of key findings on developmental stuttering in the omics context. The journey from initial linkage findings to the discovery of specific genes and pathways demonstrates the power of genomics and other omic-related avenues (Table 1).

Developmental stuttering, and in particular PDS, is undeniably a complex, heterogeneous disorder with polygenic and multifactorial influences, as highlighted by a foundational complex segregation analysis suggesting an autosomal dominant major gene effect with sex-specific penetrance and residual polygenic/multifactorial factors [4]. This complexity necessitates a holistic, multi-omic approach that moves beyond single-gene discoveries to integrate information across different biological layers. My perspective, as someone who stutters, and considering such a panorama, is that each ‘omics’ layer offers a unique window into the disorder. Currently, although such omic approaches, such as genomics and phenomics, have been applied in stuttering research, others, such as epigenomics, proteomics, and metabolomics, remain largely unexplored, offering promising directions for future studies. The current research already provides compelling examples of omic integration. For instance, the combination of neuroimaging, transcriptome data, and interactome analysis revealed novel gene intersections in stuttering [18], while exome sequencing paired with RNA sequencing in mouse models shed light on the post-translational effects of a pathogenic variant [21]. Similarly, phenomics-driven cohort building from EHRs directly informs and strengthens subsequent GWAS [5,14].

To advance research understanding, the scientific field might consider strategies and potential aspects as follows:

1. **Expanded Genomic Discovery and Validation**: Larger, more diverse, and trans-ethnic WES studies are needed to capture the full spectrum of genetic variation, including structural variants, that might contribute to PDS. Functional validation of identified variants in diverse cellular and animal models, including induced pluripotent stem cells (iPSCs) derived from individuals who stutter, is paramount to understand their biological impact.
2. **Transcriptomic and Epigenomic Profiling**: The exemplary observation that a specific variant reduced gene expression [13] or that relevant variant effects manifest post-translationally rather than transcriptionally [21] highlights the importance of going beyond DNA sequence alone. Comprehensive analyses of gene expression (transcriptomics) and epigenetic modifications (epigenomics) in relevant neural regions-such as iPSC-derived neurons, which provide a controllable human cell model for basic and translational research, and strategic tissues, including organoid models—might be relevant for understanding how genetic variants impact gene regulation and cellular function in the context of PDS.
3. **Proteomics and Metabolomics**: Investigating protein profiles (proteomics), including post-translational modifications such as phosphorylation (phosphoproteomics), alongside the comprehensive study of metabolites (metabolomics) through targeted or untargeted approaches, may provide dynamic insights into the functional consequences of genetic and transcriptomic alterations, potentially uncovering novel biomarkers or therapeutic targets. While direct proteomic and metabolomic investigations in individuals with developmental stuttering are currently limited, approaches from related human neurodevelopmental research may be adapted to probe molecular mechanisms and identify candidate biomarkers or relevant molecular and biochemical strategies in future investigations.

Building on recent advances in phosphoproteomics in humans [24,25], Kalyuzhnyy et al. [24] underscored the importance of rigorous data curation and statistical control in high-throughput studies, showing that insufficient false discovery rate correction can inflate the number of identified phosphosites. Applied to neurodevelopmental contexts, such as stuttering, this perspective suggests that implementing database-level validation and probabilistic ranking of candidate biomarkers will be crucial to avoid misleading signals and enhance reproducibility. In parallel, Ochoa et al. [25] highlighted the relevance and potential of integrating large-scale phosphoproteomic datasets with machine learning to prioritize functionally relevant phosphorylation sites, validating their impact on neuronal differentiation through chromatin-remodeling relevant proteins and related strategies. Translating this approach to stuttering, future studies could combine phosphoproteomic (in addition to other proteomic approaches), structural, regulatory, and evolutionary features to identify and functionally rank molecular markers that shape neural circuits of speech and language. Together, these complementary insights point toward a next generation of research in developmental stuttering that is more predictive, mechanistic, and translational, enabling the discovery of validated biomarkers and therapeutic targets.

The lack of available human samples or established experimental frameworks may be cited as key limitations. Furthermore, despite substantial advancements in mass spectrometry (MS) techniques, certain omic approaches—for instance, generating a comprehensive human phosphoproteome map—still face significant analytical and statistical challenges. These include the potential accumulation of false positives in public resources due to inadequate aggregation of parallel MS searches, the incomplete coverage of the phosphoproteome resulting from persistent difficulties in detecting specific phosphopeptides—particularly those from low-abundance proteins—and challenges in accurately localizing phosphorylation events [24,25]. Future studies could leverage model systems, such as iPSC-derived neurons or organoid models, in combination with advanced computational approaches, to explore molecular and biochemical mechanisms and identify candidate biomarkers in a controlled and ethically feasible manner.

4. **Integrated Phenomics and Clinical Data**: It is relevant that the meticulous phenotyping efforts, such as those leveraging EHRs and phecode analysis [5], continue to evolve. This includes integrating detailed speech-language phenotyping with neuroimaging, cognitive assessments, and behavioral data to refine PDS sub-types and enable more powerful gene-phenotype correlations. Future research might critically address limitations evident across the literature, including sample sizes, cohort bias, and variability in phenotyping approaches. As illustrated in Table 1, studies ranging from linkage scans in diverse populations and cohorts highlight the importance of larger, multi-site, and trans-ethnic investigations to capture the full genetic heterogeneity of developmental stuttering. To improve reproducibility and discovery power, future work should integrate standardized, high-resolution phenotyping strategies, such as PheRC leveraging EHRs, thereby enabling more robust multi-omics analyses and well-powered GWAS.
5. **Integrating connectomic findings**: Benito-Aragón et al. [18] reviewed neuroimaging studies implicating structural and functional alterations across widespread cortical and subcortical networks in developmental stuttering, in addition other relevant aspects regarding neuroanatomical factors and brain connectomic findings. Their synthesis highlighted consistent grey matter differences in regions such as the supplementary motor area, primary motor cortex, inferior frontal gyri, pars opercularis (Brodmann area 44), classical Broca and Wernicke areas, superior temporal gyri, insula, precuneus, basal ganglia-thalamo-cortical loops, and cerebellum, as well as changes in axonal tracts connecting perisylvian, motor, and auditory regions.

This body of evidence underscores the notion that stuttering emerges from network-level disruptions rather than isolated regional deficits. Building on this framework, future studies should aim to integrate connectomic and multi-omic data, mapping genetic and transcriptomic variation directly onto these disrupted networks. Such an approach may reveal how molecular alterations contribute to the development and maintenance of network-level dysfunctions, potentially identifying mechanistic links between relevant aspects such as gene expression, cytoskeletal organization, and functional connectivity patterns underlying persistent stuttering. By combining high-resolution neuroimaging with multi-omic profiling, the field could move towards predictive models that connect molecular etiology to observable behavioral phenotypes, offering avenues for targeted interventions and personalized therapies.

6. **Computational Biology**: Advanced computational tools and artificial intelligence will be relevant to integrate and interpret the vast, multi-layered datasets generated by omics studies and other related parameters on people who stutter, identifying complex networks, useful tools and approaches, and subtle interactions that traditional or ongoing methods might miss.
7. **Latest Investigations-From Candidate Genes to Polygenicity**: Very recently, in 2025, Polikowsky et al. [23] reported a large GWAS study of developmental stuttering, primarily leveraging data from 23andMe, analyzing nearly 100,000 cases and over one million controls. The study confirmed that stuttering has a highly polygenic architecture, identifying 57 genomic loci and validating Polygenic Risk Scores in independent cohorts. Functional analyses showed enrichment in neuronal expression, conserved regions, and enhancer elements, while genetic correlation analyses indicated overlap with autism, depression, and impaired rhythm synchronization, supporting a link between rhythm deficits and stuttering. The study also reinforced previously reported candidate genes [24].

The above-mentioned recent work provides strategic and innovative insights to dissect the molecular basis of stuttering, reinforces its neurodevelopmental and polygenic nature, and highlights promising avenues for future research, including potential translational and applied applications. In particular, combining GWAS insights with functional omics in neuronal models could help clarify how risk variants shape neural circuits and rhythmic processing. Moreover, integrating longitudinal and cross-ancestry studies will be essential to fully capture the genetic and environmental interplay underlying developmental stuttering and PDS.

Another very recent (2025) investigation, conducted by Eising et al. [22], applied WES to 85 parent-offspring trios to investigate de novo variants in developmental stuttering. This omic approach identified pathogenic or likely pathogenic variants in key genes, establishing direct genetic link between stuttering and other neurodevelopmental disorders. The authors also highlighted two genes of interest: *FLT3* (Fms Related Tyrosine Kinase 3) and *IREB2* (Iron Responsive Element Binding Protein 2). Comprehensive bioinformatic analyses, including assessments of gene expression in the developing and adult human brain, did not find links to specific brain processes or overlapping expression patterns among the twelve genes associated with monogenic forms of stuttering [25]. This lack of molecular convergence suggests significant heterogeneity in the etiological basis of stuttering. Therefore, future studies may also focus on identifying recurrent mutations in these genes in larger cohorts (in addition to the focus regarding other relevant mutations) or conducting extensive phenotypic assessments in individuals carrying these variants to functionally prove their causal role in stuttering and elucidate the underlying biological mechanisms.

8. **Other integrative approaches and strategies**: The findings of Jackson et al. [26], demonstrating that adults who stutter largely do not exhibit stuttering during private speech, highlight the central role of social perception and communicative context in the manifestation of stuttering events. This points toward a potential paradigm shift and provides a unique opportunity for multi-omic integration and multidisciplinary strategies, where omic approaches and neuroimaging data could be combined to disentangle the biological underpinnings of context-dependent fluency and PDS. For instance, future studies could leverage omics approaches to investigate molecular signatures associated with neural circuits, social-cognitive networks, and behavioral processes engaged during private versus socially oriented speech, potentially revealing pathways involved in communicative stress regulation and self-monitoring. Moreover, future research might leverage integrative multi-omic frameworks to link behavioral phenotypes with underlying molecular networks, advancing the field from descriptive behavioral observations toward mechanistic models of developmental stuttering. Such approaches could ultimately reveal context-specific biomarkers and therapeutic targets, deepening our understanding of how social and biological dimensions converge to shape speech fluency within the stuttering context.

Previous investigation has also highlighted how prenatal insults, particularly maternal stress, can exert sex-specific effects on neurodevelopment via placental signaling, with males showing heightened vulnerability to disorders such as autism and dyslexia. Stuttering has likewise been mentioned to be related to exhibit sex-biased patterns [27]. By identifying X-linked genes such as O-linked N-acetylglucosamine transferase as causal mediators of male-specific stress phenotypes, the placenta as a critical interface in neurodevelopmental programming was highlighted [27]. Translating these insights into developmental stuttering research may open new avenues for multi-omic integration, where datasets from placental biology could be linked with neuroimaging and omic profiles of speech-related parameters in large-scale studies of individuals. Such an approach could disentangle how early-life stressors and sex-specific molecular mechanisms interact to shape vulnerability or resilience to stuttering. Future studies that adopt this systems-level perspective may uncover biomarkers of risk that are not only mechanistically informative but also predictive of developmental trajectories, ultimately guiding precision strategies for prevention and intervention. Furthermore, links between developmental stuttering and neuropsychiatric conditions naturally raise questions about whether pharmacological or nutritional approaches commonly used in these disorders might influence stuttering. While some omics-based investigations have not yet directly addressed these possibilities, future multi-omics studies may provide mechanistic insights relevant to such approaches.

### Bibliometric Analysis of Research Trends

**Figure 2:**
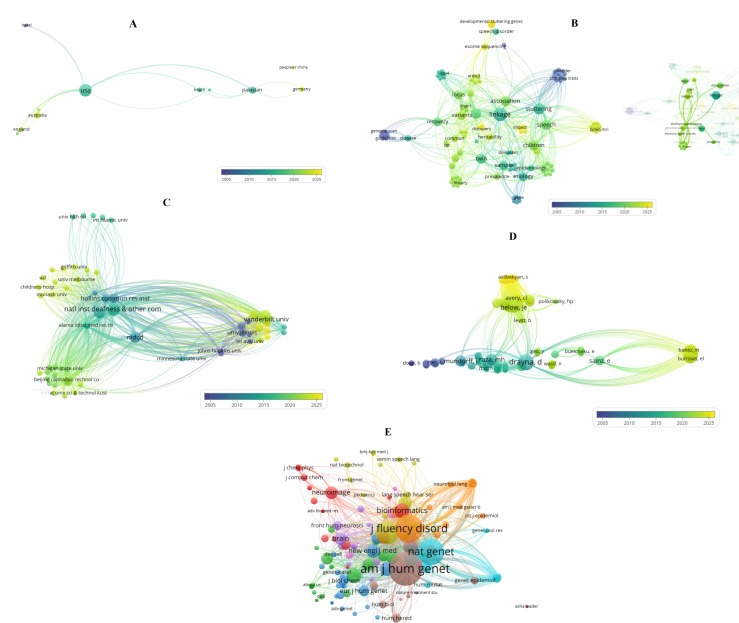
Bibliometric analysis and integrative mapping of the developmental-stuttering literature with emphasis on omics (**Panels A**–**E**). Node color represents the average publication year (blue = earlier, yellow = recent), node size encodes the relevant weight metric for each analysis, and edge thickness indicates relationship strength. (**Panel A**): co-authorship by country. (**Panel B**) keyword co-occurrence. (**Panel C**) institutional citation impact. (**Panel D**) bibliographic coupling of authors. (**Panel E**): co-citation of sources. Together these maps reveal global hubs, thematic clusters, institutional influence, intellectual lineages, and core journals shaping omics research in developmental stuttering.

I also performed a bibliometric analysis using the Web of Science Core Collection, providing a comprehensive overview of scientific topics and key aspects related to stuttering within the omics context. Method details of this analysis are presented in Supplementary Materials File S1. Using VOSviewer software (version 1.6.20; Leiden, The Netherlands) [28] and a systematic examination of the publications, I generated Figure 2A–E, which include multiple graphs and illustrate the results indicating multiple research dimensions as well as emerging and relevant trends in this field.

Figure panels 2A–E provide a compact, complementary view of who produces the stuttering literature (A, C), what topics and terms dominate (B), how authors cluster by shared reference use (D), and which journals and sources form the intellectual backbone of the field (E). Interpreting those networks together highlights a field dominated by genetics- and multidisciplinary-oriented inquiry, centered in some institutional hubs, and now showing a recent uptick in sequencing and computational-methods work.

The United States dominates as the main producer and collaborative hub, linking strongly with countries such as Australia, England, Brazil, Israel, and Pakistan. More recent activity is visible in countries like Germany, which appear in warmer tones, while others such as Israel reflect an earlier wave of studies. Emerging contributors, including Brazil and Pakistan, play bridging roles that could diversify genetic datasets (Figure 2A). These patterns suggest that omics progress will require multinational consortia that expand beyond traditional hubs, enhancing sample size and ancestral diversity.

The keyword map highlights different interconnected thematic clusters. One centers on genetics and genomics, with terms such as linkage, association, heritability, exome sequencing, twin, and variants, reflecting the classical gene-discovery trajectory. The other emphasizes clinical and neurophenotypic dimensions, with terms such as stuttering, speech, children, magnetic resonance imaging, and prevalence. Bridging keywords connect these domains, underscoring the importance of precise phenotyping in omics studies. Yet terms such as transcriptomics, epigenetics, proteomics, or single-cell remain sparse, pointing to a critical gap (Figure 2B). For omics to move from variant discovery to mechanism, the field must integrate multi-omics sampling with harmonized behavioral and neuroimaging data, supported by computational pipelines that connect genotype, brain, and phenotype.

A few institutions dominate in terms of citation impact, including national research centers and specialized universities. These hubs form dense, reciprocal connections and concentrate resources for sequencing and computational analysis. The pattern highlights both the strength and limitation of centralization: while it accelerates progress, it risks overreliance on a small number of centers. Moving forward, consortium frameworks that distribute protocols, raw data, and training from established hubs to emerging groups are crucial to broaden the base of omics research (Figure 2C).

Author-level analysis reveals distinct intellectual lineages. One major cluster is centered on long-standing genetics research programs, while other clusters reflect neuroimaging, clinical epidemiology, or emerging sequencing efforts. The differences in reference use suggest partially isolated subfields (Figure 2D). Omics integration will require deliberate cross-training and the construction of shared reference frameworks so that genetics, neuroimaging, and clinical researchers converge around common datasets and standards.

The co-citation map identifies the journals that form the intellectual foundation of the field such as: Journal of Fluency Disorders for clinical research, Nature Genetics and AJHG for genetics, NeuroImage and Brain for neuroimaging, and Bioinformatics for computational approaches. The dense interconnections among these journals illustrate a multidisciplinary landscape (Figure 2E). The presence of Bioinformatics as a central node signals the growing importance of computational methods in linking genetic and neuroimaging data.

Taken together, such set of information shows a field transitioning from classical genetics and twin studies toward sequencing-based discovery and computational integration with neuroimaging. To capitalize on this momentum, several priorities emerge such as: build a global stuttering omics consortium with standardized behavioral and imaging phenotyping; secure harmonized consent for multi-omics data collection and sharing; expand efforts into transcriptomics, epigenomics, and proteomics, as well other potential omic strategies; and ensure capacity building in emerging research nodes. The current literature (Figure 2) also highlights the need for expanded multi-omic research and the initiation of new, larger-scale studies, particularly in underrepresented omic areas. Finally, integrative pipelines and shared training between geneticists, neuroimagers, and clinical scientists as well as multidisciplinary integration are essential for translating omics findings into mechanistic insights about stuttering biology and related applied aspects.

## 6. Impact on Individuals, Policy, and Society

For individuals who stutter, these scientific insights carry profound significance. Understanding the biological underpinnings of stuttering can challenge long-standing misconceptions, reduce self-blame, and foster a sense of validation. It moves the narrative from a perceived psychological “flaw” to a genuine neurobiological condition, deserving of scientific inquiry and support. The identification of specific genes and pathways opens doors for the development of targeted, personalized therapeutic interventions that could complement or enhance existing speech therapy approaches.

Beyond clinical applications, the robust scientific evidence generated by multi-omic research on developmental stuttering and PDS can be a powerful catalyst for societal change. It might inform policy and regulatory frameworks to improve accessibility, support, and the rights of people who stutter, needs that are often unrecognized or inadequately addressed. This includes advocating for better healthcare coverage for interventions, ensuring reasonable accommodations in educational and professional settings, and combating discrimination. A deeper biological understanding lends credibility to the call for PDS to be explicitly recognized as a disability, where appropriate, ensuring legal protections and equitable opportunities. By clearly demonstrating the context or biological basis of stuttering, it is possible to empower individuals, educate the public, and drive policy changes that create a more inclusive and understanding society for all people who stutter.

## 7. Concluding Remarks

The multi-omic era offers an unprecedented opportunity to dissect the intricate genetic and biological mechanisms underlying developmental stuttering. From identifying broad chromosomal linkages and specific gene variants, such those related to lysosomal trafficking, intracellular transport, and neuronal integrity, to characterizing the comprehensive phenome of stuttering, research is steadily illuminating this complex disorder. The integration of genomics, transcriptomics, and phenomics, coupled with advanced computational approaches and other relevant omic strategies, promises to yield a more comprehensive understanding of PDS at a systemic level. The bibliometric analysis and integrative mapping highlight a field where progress will depend on global collaboration, multi-omic expansion, and deliberate convergence across multidisciplinary lineages and relevant related aspects concerning stuttering research. These scientific breakthroughs are not just academic achievements; they are crucial steps toward de-stigmatizing stuttering, developing personalized treatments, and advocating for a more equitable and supportive society for people who stutter.

## Figures and Tables

**Table 1:** Examples of research observations, molecular features, and genetic findings regarding developmental stuttering in the omics context.

Omics Layer and Method/Technology	Key Findings	Biological/Clinical Relevance for Human Stuttering	Sample/Population/Reference
Transcriptomics/Connectomics: Functional connectivity MRI (fcMRI)/Graph theory; Transcriptome spatial similarity analysis (AHBA); GO/Interactome analysis.	Gene *GNPTG* (mannose-6-phosphate lysosomal targeting pathway) co-localized significantly with the stuttering cortical network. Genes related to neurofilament cytoskeleton organization (*NEFH*, *NEFL*, *INA*) were highly enriched. Intermediary genes *CDK5* and *SNCA* act as functional links between lysosomal and neurofilament pathways.	Supports that stuttering is linked to lysosomal dysfunction which has deleterious effects on the neurofilament organization of speech neuronal circuits. The auditory-motor integration network is highly vulnerable to GNPTG-lysosomal malfunctioning.	Adult *Homo sapiens* (AHBA transcriptome data); Adults and CWS and NFC (*Homo sapiens*) (fcMRI data)/[18]
Genomics/Exome Sequencing.	A novel heterozygous exonic variant (c.322G > A) in *NAGPA* segregated with the phenotype. Other heterozygous exonic variants were found in *RIMS2* and *XYLT1* in severely affected members, and *ATP13A2* (*PARK9*) was found via pathway analysis. Context: These variants suggest contributions from the lysosomal pathway (*NAGPA*, *ATP13A2*) and potentially dopamine signaling (*RIMS2*, *ATP13A2*).	Stuttering likely arises from the combined effect of gene variants at multiple loci (genetic heterogeneity). The findings suggest a role for lysosomal dysfunction and, for the first time, a likely role of dopamine signaling in stuttering.	A multiplex *Homo sapiens* family pedigree (*n* = 27 samples for sequencing; *n* = 21 additional family members for validation/[19]
Genomics/Genome-wide linkage scan; SNP genotyping; Microsatellite fine mapping.	A novel linkage locus for PDS was identified at chromosome 10q21. Significant evidence of linkage was found under an autosomal dominant model of inheritance, generating a combined maximum multipoint LOD score of 4.28 (at marker D10S1790).	Identification of a novel genetic mechanism and locus (10q21) in an admixed population, consistent with the high genetic heterogeneity of PDS. The mode of inheritance observed was dominant, contrasting with recessive models often found in consanguineous populations.	43 unrelated Brazilian families (*Homo sapiens*) recruited in São Paulo State, Brazil (312 total individuals); linkage signal was driven by two multiplex families (BRPD47 and BRPD50)/[12]
Genomics/WES; De novo variant identification (Trio design).	A pathogenic de novo stop-gain variant was found in *SPTBN1*. Likely pathogenic de novo missense variants were identified in *PRPF8*, *TRIO*, and *ZBTB7A*. Context: These are rare variants in genes previously linked to various neurodevelopmental disorders.	Provides the first direct genetic link between stuttering (including both persistent and transient forms) and other neurodevelopmental disorders (e.g., speech delay and aphasia). Results support etiological heterogeneity, as the associated genes do not converge onto a shared biological pathway or expression pattern.	85 independent parent–child trios (*Homo sapiens*) (children with transient or persistent stuttering; unaffected parents)/[22]
Genomics/Linkage Analysis; DNA Sequencing (Exons, Exon-Intron Boundaries, Promoter regions).	Pathogenic mutations (missense, stop-gain, deletion, duplication) were identified in three genes: *GNPTAB*, *GNPTG*, and *NAGPA*. These genes encode enzymes responsible for generating the M6P signal, central to the lysosomal enzyme–targeting pathway.	Susceptibility to nonsyndromic, PDS is associated with genetic variations in the lysosomal metabolism pathway. This suggests that deficits in lysosomal enzyme targeting may specifically affect the neural structures and motor functions required for fluent speech.	Consanguineous Pakistani families and unrelated affected subjects from Pakistan, North America, and Britain (*Homo sapiens* adults with persistent stuttering)/[15]
Genomics/Exome Sequencing; Molecular Dynamics Simulation; MRI ( DWI, QSM).	An ultra-rare missense variant (c.808C>T, p.Pro270Ser) in *PPID*, encoding the chaperone protein *CYP40*, segregated with stuttering. Context: The mutation disrupts the TPR2 domain and is predicted to inhibit Cyp40 binding to Hsp90.	Implicates a chaperone protein in PDS pathogenesis. Findings support disruption of the CSTC network. DWI of the knock-in model revealed significant microstructural changes (increased ADC, AD, RD) in the left corticospinal tract.	Four-generation Australian Caucasian family (*Homo sapiens*); Humanized knock-in *Ppid* p.Pro270Ser mice (*Mus musculus*)/[21]
Genomics/ GWAS; Meta-analyses (sex and ancestry-specific/combined); Genetic correlation; MR.	Identification of 57 unique loci associated with stuttering, mapping to 48 unique genes. Sentinel hits included *VRK2*, *CAMTA1* (European ancestry [*EUR*] males), and *SLC39A8*, *DCC* (*EUR* females). Context: *VRK2* is implicated in musical beat synchronization, providing genetic support for the Atypical Rhythm Risk Hypothesis.	Stuttering risk is highly polygenic and genetically complex, exhibiting sexual dimorphism. Revealed shared molecular underpinnings (genetic correlation) between stuttering and impaired beat synchronization, ASD, and depression. Enrichment analysis showed variants are active in neurons and brain regions (frontal cortex, basal ganglia) related to speech motor planning.	Over 1.1 million individuals (*Homo sapiens*) (99,776 cases/1,023,243 controls) from 23andMe (self-reported stuttering), stratified by sex and ancestry (*EUR*, African ancestry [*AFR*], East Asian Ancestry [*EAS*], American/Admixed Americana Ancestry [*AMR*])/[23]
Genomics/GWAS; Meta-analysis; Expression Quantitative Trait Locus (eQTL) mapping.	Identification of one GWS protective variant (rs113284510) in an intronic/genic upstream region of *SSUH2*. Context: The protective allele (T) acts as an *eQTL*, reducing *SSUH2* expression in esophagus-muscularis tissue, and increasing *CAV3* expression in tibial artery tissue.	Stuttering risk is highly polygenic and complex. Findings suggest involvement of genes related to structural organization, nervous system development, and neurogenesis. Preliminary support for shared genetic liability with ASD.	16,461 total individuals (*Homo sapiens*) (2130 cases: International Stuttering Project [ISP] clinically ascertained cases + Add Health self-reported cases; 14,331 controls). Trans-ancestry/Multiethnic cohort/[13]
Clinical Phenome/(EHR data mining; Phenotype Code (Phecode) enrichment analysis; Machine Learning ([PheRC]/Classification and Regression Tree).	Identification of 38 significantly enriched phecodes (comorbidities). Key enriched phecodes included developmental Delays (315), speech and language disorder (315.2), sleep disorders, hearing loss, atopic triad (allergies/dermatitis), neurological deficits (including aphasia/speech disturbance), and atypical weight regulation.	Established a valid, multi-step method (keyword search, text-mining, manual review) for identifying developmental stuttering cases in EHRs. Comorbidity analysis confirmed known associations and suggested novel shared etiology (e.g., with infections, body weight issues). The resultant PheRC facilitates future high-powered genetic etiology studies (GWAS).	1143 confirmed developmental stuttering cases (*Homo sapiens*); EHR data from Vanderbilt University Medical Center Synthetic Derivative (VUMC SD, ~2.8 million records)/[5]
Genomics/WES; Dideoxy sequencing; Yeast two-hybrid (*Y2H*) assay; Functional assembly assay (Immunoblotting).	Rare coding variants, including two specific heterozygous variants (c.1549G>A, p.Val517Ile and c.2401G>A, p.Glu801Lys), were identified and co-segregated with stuttering in *AP4E1*. Context: *AP4E1* encodes the epsilon subunit of the Adaptor protein complex 4 (*AP-4*) complex, which is involved in protein sorting at the Trans-Golgi Network (*TGN*). The *AP-4* complex (specifically its m4 subunit) was shown to physically interact with *NAGPA* (a previously associated stuttering gene).	Implicates deficits in intracellular trafficking and the endosomal transport system in PDS. Establishes a direct functional link between the newly identified gene (*AP4E1*) and the previously established lysosomal pathway genes (*NAGPA*, *GNPTAB*, *GNPTG*). Stuttering in heterozygotes is a non-syndromic presentation, lacking the severe symptoms of homozygous *AP4E1* deficiency.	Large Cameroonian family (*CAMST01*); Unrelated individuals with persistent stuttering from Cameroon, Pakistan, and North America (*Homo sapiens*). Human embryonic kidney 293 cells (*HEK-293 cells*) (for biochemical assays)/[17]
Genomics/Targeted Sequencing (Exons and Intronic flanks).	Rare non-synonymous coding variants in *GNPTAB*, *GNPTG*, and *NAGPA* were significantly enriched in cases (16% incidence vs. 7% background rate). Context: The variants found in stuttering subjects (92.6% missense substitutions) suggest a less severe defect in the lysosomal enzyme-targeting pathway compared to the severe LoF mutations found in Mucolipidosis (ML II/III).	Confirms that genetic variants in the lysosomal enzyme-targeting pathway contribute significantly to non-syndromic PDS (estimated 16% of cases). The difference in mutation type (missense vs. frameshift/stop-gain) explains why stuttering patients do not exhibit ML II/III symptoms.	1013 unrelated individuals with non-syndromic PDS (*Homo sapiens*) from worldwide cohorts (NAF, England, PKST, STCR, BRCS)/[16]
Genomics/Genome-wide linkage scan; SNP genotyping; Microsatellite fine mapping.	Identification of an autosomal recessive stuttering locus on chromosome 3q13.2–3q13.33. Significant linkage was confirmed with a maximum two-point LOD score of 4.23. Context: The region spans 3.24 Mb and contains 46 known/predicted genes, including *DRD3* (which showed no variation upon sequencing).	Identification of a highly significant genetic locus for PDS, supporting the substantial heritability of the disorder and demonstrating the power of using highly consanguineous families for complex trait linkage analysis.	A newly ascertained consanguineous Pakistani family (PKST77) with persistent stuttering (*Homo sapiens*)/[10]
Genomics/Genome-Wide Linkage Scan; Microsatellite and *SNP* genotyping; Two-locus analysis (Superlink).	Identification of multiple linkage loci (2p, 3p, 3q, 14q, and two on 15q). Context: The strongest combined evidence came from the 2p + 15q two-locus model, achieving LOD scores up to 6.57, supporting a complex, polygenic model.	Provides strong evidence for polygenic inheritance and locus heterogeneity in PDS. This mechanism explains the large number of affected individuals in a family without clear consanguinity. The affected individuals are typically multilingual stutterers.	Large Cameroonian family pedigree (CAMST01, 71 individuals, at least 33 affected) (*Homo sapiens*)/[11]
Genomics/WES; Sanger Sequencing.	A homozygous splice site variant (c.916 + 1G > A) in *ARMC3* was identified. Context: This LoF variant causes skipping of exon-8, predicted to lead to NMD, and significantly alters the protein’s folding and its interaction with MYCBPAP.	Implicates the novel Armadillo repeat (*ARM*) gene family and potential dysfunction in the Wnt signaling pathway in non-syndromic PDS. *ARMC3* is highly expressed in brain regions critical for motor function and emotion, such as the basal ganglia and cerebellum.	A consanguineous Pashtun family of Pakistani origin (*Homo sapiens*) with autosomal recessive PDS (two affected individuals sequenced) [20]
Genomics/*GWAS*; Phenome Risk Classification (Phenome Machine Learning [PheML])/Classification and Regression Tree Model.	Identification of genome-wide significant variants: rs12613255 (near *CYRIA*) in EUR ancestry, and rs7837758 (intronic in *ZMAT4*) in *AFR* ancestry. Context: Both genes are highly expressed in CNS regions, including the cerebral cortex and basal ganglia.	Successful population-based GWAS for developmental stuttering, confirming the risk is highly polygenic and genetically complex. The findings support a genetic etiology related to CNS function and neural circuits controlling speech motor planning. Validation through PRS confirms the model captures the genetic liability of clinically ascertained stuttering cases.	9239 PheML-imputed affected individuals (*Homo sapiens*) from the BioVU EHR-linked biorepository, analyzed in ancestry-stratified cohorts [14]
Genomics/Genome-Wide Linkage Scan; Non-Parametric Linkage Analysis; Fine mapping.	Evidence for a predisposing locus on chromosome 18 (18p and proximal 18q). Best overall Non-Parametric Linkage statistic (NPL) (Z-log odds ratio statistic [Zlr]) score was 5.143 at marker D18S78. Context: Candidate genes include the *CDH2* gene and the desmoglein/desmoc olin family, suggesting cell adhesion/intercellular communication deficits.	Suggests chromosome 18 harbors a key locus contributing to PDS. The implicated pathways (cell adhesion) may affect neurons involved in speech production. Data supports locus heterogeneity and an incomplete dominant inheritance pattern.	68 outbred families (226 individuals, 188 affected) from North America and Great Britain (*Homo sapiens*); subjects displayed persistent stuttering (>=4% dysfluencies) [7]
Genomics/WES; Sanger sequencing; Luciferase reporter assay (Functional validation).	Rare coding variants in *IFNAR1* (including Leu552Pro, Lys428Gln, Gly301Glu, and Pro335del) cosegregated with stuttering. Functional studies showed that three of these variants significantly impaired Type IIFN signaling (Janus kinase-signal transducer and activator of transcription [JAK-STAT] pathway activation).	Suggests *IFNAR1* is a novel pathogenic gene for *PDS*, particularly in the Chinese population. Impaired Type I *IFN* signaling links stuttering to underlying neurodegenerative events, abnormal autophagy, and lysosomal dysfunction.	10 independent PDS families and 84 sporadic PDS cases (DNA samples) from the Chinese population (*Homo sapiens*); HEK-293 cells (for in vitro functional assays) [2]
Genomics/Genome-wide Linkage Scan; High-density SNP genotyping (110K SNP array).	Genomewide-significant sex-specific linkage signals: Chromosome 21 (LOD 4.5) in female-only data; Chromosome 7 (LOD 2.99) in male-only data. Context: Conditional analysis supported the established Chromosome 12 locus and implicated Chromosome 2 (193 cM) through interactions.	Strong support for sexual dimorphism in the genetic architecture of stuttering. Suggests overlapping genetic liability with Autism and SLI, particularly in the Chromosome 7 and 2 regions.	100 families of European descent (*Homo sapiens*) (US, Sweden, Israel), including 252 individuals with persistent stuttering and 45 recovered cases [8]
Genomics/Linkage Analysis; Association Analysis (Transmission Disequilibrium Test [*TDT*], Family-Based Association Test [*FBAT*]); Meta-analysis (GSMA-related analysis).	Nominal linkage peaks found on Chromosomes 3, 13, and 15 (Hutterites). Highest NPL all peak was on Chromosome 13 at 52.6 cM (*p* = 0.012), which overlapped with suggestive TDT and FBAT signals. Meta-analysis highlighted Chromosomes 2 and 5.	Supports that stuttering is a polygenic disorder. The Chromosome 13 locus suggests shared genetic susceptibility with other neurodevelopmental phenotypes, including SLI, autism, and Tourette’s syndrome.	40 genotyped individuals (*Homo sapiens*) who had ever stuttered (persistent and recovered cases) from a large Hutterite founder population; meta-analysis included outbred Caucasian families [9]

**Abbreviations**: AD: Axial Diffusivity; ADC: Apparent Diffusion Coefficient; AFR: African ancestry; AHBA: Allen Human Brain Atlas; AMR: American/Admixed Americana Ancestry; AP-4: Adaptor protein complex 4; *AP4E1*: Adaptor-related protein complex 4, epsilon 1 subunit gene; ARM: Armadillo repeat; *ARMC3*: Armadillo Repeat Containing 3 gene; ASD: Autism Spectrum Disorder; *ATP13A2*: ATPase type 13A2 gene; BioVU: Vanderbilt University Medical Center’s EHR-linked biorepository; BRCS: Brazilian stuttering cases; CAMST01: Cameroonian Stuttering family 01 pedigree identifier; *CAMTA1*: Calmodulin binding transcription activator 1 gene; *CAV3*: Caveolin 3 gene; *CDH2*: Neuronal cadherin 2 gene; *CDK5*: Cyclin-dependent kinase 5 gene; cM: Centimorgan (unit of genetic distance); CNS: Central Nervous System; CSTC: Cortico-striatal-thalamo-cortical (circuit); CWS: Children Who Stutter; *CYP40*: Cyclophilin-40 protein; *CYRIA*: Gene CYFIP-related Rac1 interactor A; *DCC*: Netrin receptor DCC gene; *DRD3*: Dopamine Receptor D3 gene; DWI: Diffusion-weighted Magnetic Resonance Imaging; EAS: East Asian Ancestry; EHR: Electronic Health Record; eQTL: Expression Quantitative Trait Locus; ESP: Exome Sequencing Project; EUR: European ancestry; FBAT: Family-Based Association Test; fcMRI: Functional connectivity Magnetic Resonance Imaging; *GNPTAB*: N-acetylglucosamine-1-phosphate transferase gene encoding the subunits alpha and beta; *GNPTG*: N-acetylglucosamine-1-phosphotransferase gamma subunit gene; GO: Gene Ontology; GSMA: Genome Search Meta-Analysis; GWAS: Genome-Wide Association Study; GWS: Genome-Wide Significant Signal; HEK-293 cells: Human embryonic kidney 293 cells; *Homo sapiens*: Human species; *Hsp90*: Heat shock protein 90; ICD: International Classification of Diseases; IFN: Interferon; IFN-β: Interferon-beta; *IFNAR1*: Interferon-alpha/beta receptor 1 gene; *IGF2R*: Insulin-like growth factor 2 receptor gene; *INA*: Internexin neuronal intermediate filament protein alpha gene; ISP: International Stuttering Project; JAK-STAT: Janus kinase-signal transducer and activator of transcription; LOD: Logarithm of the Odds score; LoF: Loss of Function; M6P: Mannose-6-phosphate; Mb: Megabase (unit of physical distance on DNA); Mbp: Megabase pair (physical distance on chromosome); ML II/III: Mucolipidosis types II and III (lysosomal storage disorders); MR: Mendelian Randomization; MRI: Magnetic Resonance Imaging; *Mus musculus*: Mouse species; *MYCBPAP*: MYCBP Associated Protein; NAF: North American Families; *NAGPA*: N-acetylglucosamine-1-phosphodiester alpha-N-acetylglucosaminidase gene; *NEFH*: Neurofilament heavy gene; *NEFL*: Neurofilament light gene; NFC: Normally Fluent Controls; NIH: National Institutes of Health; NMD: Nonsense Mediated Decay; NPL: Non-Parametric Linkage statistic; *PARK9*: Parkinson’s disease gene 9; PDS: Persistent Developmental Stuttering; Phecode: Phenotype Code; PheML: Phenome Machine Learning; PheRC: Phenome Risk Classifier; PKST: Pakistani stuttering; *PPID*: Peptidyl-prolyl isomerase D gene; *PRPF8*: Pre-mRNA-processing-factor 8 gene; PRS: Polygenic Risk Score; QSM: Quantitative Susceptibility Mapping; RD: Radial Diffusivity; *RIMS2*: Regulatory molecule in synaptic vesicle exocytosis 2 gene; *SLC39A8*: Solute carrier family 39 member 8 gene; SLI: Specific Language Impairment; *SNCA*: Alpha-synuclein gene; SNP: Single Nucleotide Polymorphism; *SPTBN1*: Spectrin beta, non-erythrocytic 1 gene; *SSUH2*: SSUH2 gene; SSI-3: Stuttering Severity Instrument, 3rd edition; STCR: Stuttering Cameroonian random (cases); TDT: Transmission Disequilibrium Test; TGN: Trans-Golgi Network; TPR2: Tetratricopeptide repeat 2; *TRIO*: Trio Rho guanine nucleotide exchange factor gene; *VRK2*: Vaccinia related kinase 2 gene; VUMC SD: Vanderbilt University Medical Center Synthetic Derivative; WES: Whole-Exome Sequencing; Wnt: Wingless and Int-1 (signaling pathway); *XYLT1*: Xylosyltransferase 1 gene; Y2H: Yeast two-hybrid; *ZBTB7A*: Zinc finger and BTB domain containing 7A gene; *ZMAT4*: Zinc finger matrin-type protein 4 gene; Zlr: Z-log odds ratio statistic.

## Data Availability

No new data were created or analyzed in this study. Data sharing is not applicable to this article.

## Supplementary Materials

The following supporting information can be downloaded at: https://www.mdpi.com/article/10.3390/life15101630/s1. Method details of this analysis are presented in Supplementary Materials File S1.
