# Supplementary material for: Unraveling Stuttering Through a Multi-Omics Lens
Source: Life (Basel). 2025 Oct 19;15(10):1630. doi: 10.3390/life15101630 (PMC12565724; doi:10.3390/life15101630)
Supplement: Supplementary file 1 [file life-15-01630-s001.zip › life-3922042-supplementary.pdf]

Supplementary File S1

Unraveling Stuttering Through a Multi-Omics Lens

Deyvid Novaes Marques *

Department of Genetics, University of São Paulo (USP), Piracicaba, São Paulo (SP), Brazil
* Correspondence: deyvidnovaes@gmail.com

To develop a comprehensive perspective on stuttering in the context of omics research and to conduct a bibliometric analysis, a strategic literature search was conducted in the Web of Science Core Collection. The search combined terms related to stuttering—including "stuttering," "person who stutters," "stuttering disorder," and "stutter"—with a broad range of omics-related terms, such as genome, genomics, exome, omic, omics, transcriptomics, multi-omics, and related variants. This approach aimed to capture publications that specifically addressed stuttering while encompassing diverse omics disciplines, reflecting the multi-layered biological data inherent to these studies, including integrative multi-omics analyses.

The search was performed using the topic field, which searches within titles, abstracts, and author keywords. This ensured that retrieved articles had stuttering and omics concepts as central elements, maximizing the relevance of the results. The use of strategic operators between different terms expanded the pool of relevant literature, accounting for variations in terminology across different studies and avoiding the omission of important research. No publication year limitations were applied, allowing the identification of both foundational studies and the most recent advancements, providing a complete temporal overview of the field.

This strategy generated 172 publications, which were then manually reviewed. During this selection process, review articles were excluded, retaining only original research focused on developmental stuttering or persistent stuttering. Following this manual curation, the selected records were exported and analyzed using VOSviewer (version 1.6.20, Leiden, The Netherlands) [28]. This software was chosen for its ability

to visualize bibliometric networks, form clusters, and explore connections between publications, enabling a structured and detailed analysis of trends, research gaps, and the current state of stuttering studies within the omics context.
